# Rhabdomyolysis Associated With Excess Pine Bark Extract: A Case Report

**DOI:** 10.7759/cureus.93956

**Published:** 2025-10-06

**Authors:** Arya Kermanshah, Sana Master, Tea Kaceli, Ashmeet Bedi, Garine Kalaydjian

**Affiliations:** 1 Internal Medicine, St. John's Riverside Hospital, Yonkers, USA; 2 Medicine, Lake Erie College of Osteopathic Medicine, Erie, USA

**Keywords:** acute kidney injury, case report, dietary supplements, muscle toxicity, rhabdomyolysis

## Abstract

Rhabdomyolysis can cause complications such as acute kidney injury (AKI), with common etiologies including strenuous exercise, trauma, medications, and toxins. Given that creatine kinase (CK) levels of about 100,000 U/L are rare, this level of elevation may indicate the presence of an underlying contributor, such as excessive pine bark extract intake, emphasizing the importance of taking a thorough patient history to identify factors that may otherwise be overlooked. We present a case of a 35‑year‑old previously healthy male who presented with diffuse myalgia, soreness, and tea‑colored urine two days after lifting furniture for a friend. The physical examination revealed mild tenderness and edema in his upper extremity muscles. Laboratory studies showed an initial CK of 154,000 U/L, aspartate aminotransferase (AST) of 2,400 U/L, alanine aminotransferase (ALT) of 700 U/L, and normal renal function (creatinine: 0.7-0.8 mg/dL throughout admission). Urine toxicology, viral serologies, and antinuclear antibody (ANA) were negative. During a subsequent interview, the patient disclosed consuming over-the-counter French pine bark extract in excess of the labeled recommendations. On day six of the patient’s admission, the CK level declined to 7,874 U/L; transaminase levels decreased to AST of 252 U/L and ALT of 468 U/L with aggressive IV hydration. There was no evidence of kidney injury during the hospital course. This case suggests a potential association between excessive pine bark extract consumption and severe rhabdomyolysis triggered by minimal physical activity, supported by a Naranjo score of 3. Clinicians should inquire about dietary supplement use when CK levels are disproportionately elevated relative to the inciting event.

## Introduction

Rhabdomyolysis occurs when the release of toxic intracellular components, such as proteins and electrolytes, interferes with the body's homeostatic functions in the context of severe muscle damage. Particularly in cases of severe muscular necrosis, symptoms can vary from mild muscle weakness and soreness to severe complications such as acute kidney injury (AKI). A creatine kinase (CK) level five times the upper limit of normal or greater than 1,500 U/L is usually considered a hallmark finding, with severe instances frequently exceeding 10,000 U/L [1]. However, even in high-intensity training, which poses an increased risk for rhabdomyolysis compared to other exertive activities, CK levels rarely exceed 100,000 U/L [2,3]. The highest reported CK level in medical literature in the setting of rhabdomyolysis is 1,778,856 U/L in a pediatric patient and 1,353,105 U/L in an adult patient [4,5].

More than 70% of Americans report taking dietary supplements regularly, with similar patterns exhibited worldwide [6,7]. One particular supplement, pine bark extract, is used for its antioxidant and cardiometabolic benefits [8]. Prior research aimed to determine its efficacy in managing elevated blood pressure and its potential role in reducing vasoconstrictive molecules, such as endothelin [9].

At recommended dosages, pine bark extract has a favorable safety profile, with minimal side effects and no evidence of severe muscle toxicity, as supported by comprehensive systematic reviews and meta-analyses [10,11]. However, an in vitro study suggests that high doses (≥ 40-100 μg/mL) can cause apoptosis, calcium dysregulation, and mitochondrial malfunction, potentially leading to myotoxicity [12,13]. There have been no reports of pine bark extract-associated rhabdomyolysis in the medical literature, despite these results.

This case report was prepared in accordance with the CARE (CAse REport) guidelines for case reports [14].

## Case presentation

A 35-year-old male with no prior medical history, prescribed medications, or substance use presented to the emergency department with diffuse myalgias and dark, tea-colored urine. He was assisting a friend in transporting furniture two days prior. He denied heat exposure, extended immobility, trauma, dehydration, or viral prodrome, but endorsed exclusively drinking unspecified pine bark extract as a dietary supplement. Upon arrival, he was afebrile, hemodynamically stable, and fully oriented, exhibiting only mild somnolence. Physical examination revealed proximal upper extremity pain and edema, but no rash or focal neurological impairments. Cardiopulmonary and abdominal tests were unremarkable. Family history was non-contributory for any metabolic or muscular disorders.

On hospital day (HD) 1, laboratory tests indicated a significantly increased CK level of 154,112 U/L, aspartate aminotransferase (AST) of 2,357 U/L, and alanine aminotransferase (ALT) of 697 U/L. Total bilirubin was 1.5 mg/dL, and alkaline phosphatase was normal. Renal function remained stable during hospitalization, with creatinine levels ranging between 0.7 and 0.8 mg/dL and an estimated glomerular filtration rate (eGFR) greater than 110 mL/min/1.73 m². There were no abnormalities in the electrolytes. Urinalysis findings revealed 3+ blood, 3+ protein, and 19 red blood cells per high-power field, with a specific gravity of 1.011 and no ketone values.

Additional testing revealed negative urine toxicology and acetaminophen levels, as well as negative hepatitis A, B, and C serologies (with hepatitis B surface antibody reactive due to prior vaccination). Additionally, testing was negative for human immunodeficiency virus (HIV), coronavirus (SARS-CoV-2), influenza, and respiratory syncytial virus (RSV). An antinuclear antibody (ANA) was negative on HD4. Abdominal and renal ultrasound revealed no hepatobiliary pathology and only mild fullness of the collecting system without obstruction. An electrocardiogram demonstrated a normal sinus rhythm.

During a focused follow-up interview while in the hospital, the patient revealed that he had taken excessive doses of an over-the-counter pine bark extract supplement, for "antioxidant/energy" effects. The exact product brand was unknown. He denied using any other supplements, including statins, anabolic steroids, creatine, stimulant "pre-workout" products, and any other drugs.

**Figure 1:**
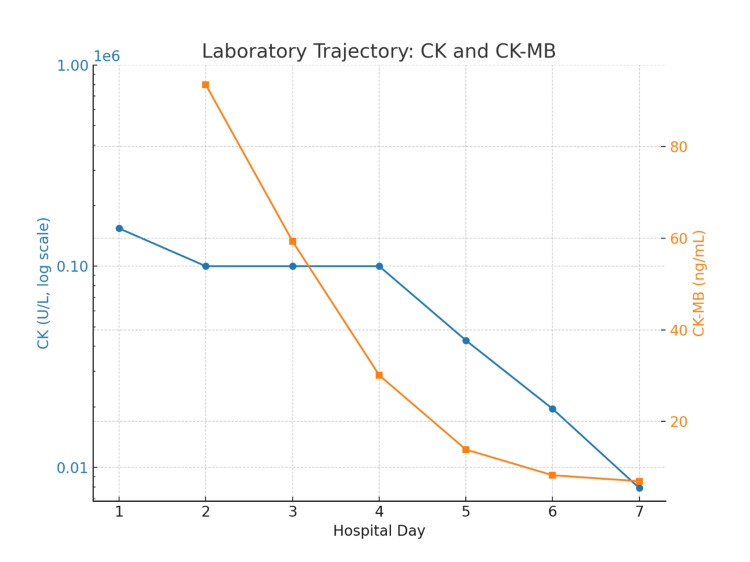
Serum creatine kinase and creatine kinase-MB during hospitalization Serum creatine kinase (CK) values (blue, log scale, left axis) demonstrated an initial peak above 150 k U/L, followed by a rapid decline after cessation of pine bark extract and initiation of aggressive intravenous fluids. Creatine kinase-MB (CK-MB) values (orange, right axis) were initially elevated at 93.4 ng/mL at presentation and showed a downward trend for the remainder of the hospitalization.

**Figure 2:**
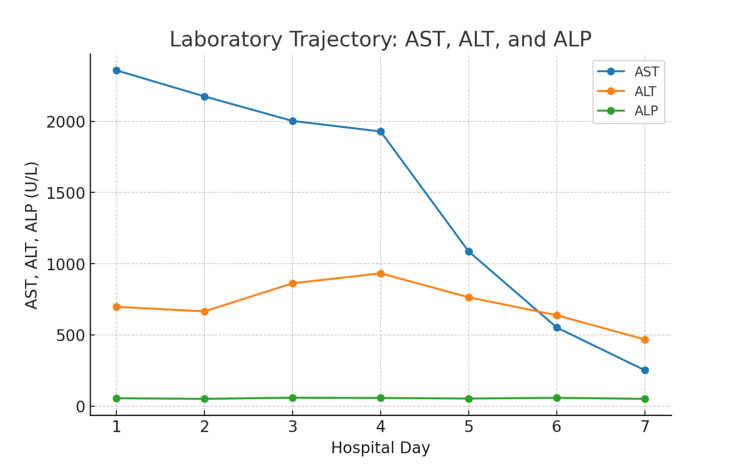
Serum transaminases during hospitalization Serum aspartate aminotransferase (AST, blue) and alanine transaminase (ALT, orange), shown on the left axis, are both elevated at presentation and declining after HD4. Alkaline phosphatase (ALP, green) remains within the normal range throughout the admission.

The supplement was discontinued, and the patient was treated with intensive intravenous isotonic fluids (first boluses, then normal saline at 125-200 mL/hr, with brief use of Lactated Ringer's (LR) (early in the course), strict intake/output monitoring, and analgesia. CK levels showed a steady decline, decreasing from over 150,000 U/L on HD1 to 100,000 U/L on HD2-3, 42,797 U/L on HD4, 19,558 U/L on HD5, and 7,900 U/L by HD6 (Figure 1). By HD6-7, transaminase levels also declined, with ALT decreasing from 697 to 468 U/L and AST from 2,357 to 252 U/L (Figure 2, with further laboratory findings given in Table 1). Urinalysis results normalized, and by HD6, the patient demonstrated stable renal function, clear urine output, and improved laboratory values. At that point, he was asymptomatic. His discharge instructions included outpatient nephrology and primary care follow-up, activity limitation for two to three weeks, and oral hydration (≥3 L/day).

**Table 1:** Serum creatine kinase (CK), aspartate aminotransferase (AST), alanine aminotransferase (ALT), creatinine (Cr), and total bilirubin (T. bili) values are shown alongside urinalysis findings Hospital day (HD) is indicated for each measurement. Red blood cells per high-power field (RBC/hpf) and specific gravity (SG) are reported for urinalysis. CK values on hospital days 2-3 exceeded the analyzer’s reporting limit and are displayed as 100,000 U/L for consistency.

Hospital Day (HD)	CK (U/L)	AST (U/L)	ALT (U/L)	Cr (mg/dL)	T. Bili (mg/dL)	Urinalysis (key findings)
HD1	154,112	2,357	697	0.8	1.5	SG 1.011; 3+ blood; 3+ protein; 19 RBC/hpf
HD2	>100,000	2174	665	0.8	1.2	Not repeated
HD3	>100,000	>2,002	862	0.7	0.7	Not repeated
HD4	>100,000	1,928	932	0.8	0.9	SG 1.007; 3+ blood; 1+ protein; 10 RBC/hpf
HD5	42,797	1,086	764	0.8	0.8	Not repeated
HD6	19,558	552	638	0.7	0.8	Not repeated
HD7	7,900	252	468	0.8	0.9	Not repeated

## Discussion

CK levels exceeding 150,000 U/L are uncommon and reflect severe skeletal muscle degradation. The release of large amounts of myoglobin and electrolytes increases the risk of AKI, hyperkalemia, and arrhythmias, predisposing to cardiac events and multiorgan failure [1]. However, the development of AKI and risk of mortality are not solely determined by CK levels, but are more closely linked to coexisting factors, such as underlying comorbidities, dehydration, and nephrotoxin exposure. In cases where these risk factors were absent and early IV hydration was initiated, patients with preserved renal function (even with markedly elevated CK levels) did not progress to AKI. This observation was supported by data from a large 2024 cohort study [15].

The patient's presentation is noteworthy due to the absence of risk factors and his demographic profile. He is a young, otherwise healthy individual with no preexisting conditions, who sought medical attention after recognizing new-onset dark urine and myalgias. The patient's health awareness and his timeliness to seek care suggest that his outcome was not the result of delayed treatment or prior medical conditions. Despite a CK peak over 150,000 U/L, this patient did not develop AKI, highlighting the protective impact of early fluid resuscitation and the need to identify risk variables other than CK level. While exertional rhabdomyolysis is a plausible explanation, the degree of CK increase seen in this case is significantly disproportionate to the reported activity. A 2023 comprehensive analysis of 772 cases found a mean CK level at presentation of around 31,500 U/L, with the highest results slightly above 100,000 U/L. Levels beyond 150,000 U/L are highly unusual, even in acute exertional instances [2,3]. These data indicate that physical activity alone is unlikely to account for the extreme CK elevation, suggesting another contributory factor.

A possible explanation is that overconsumption of pine bark extract could have decreased the threshold for myocyte damage. According to in vitro research, at supraphysiologic doses, pine bark extract causes apoptosis, interferes with mitochondrial function, and upsets calcium homeostasis [12,13]. By sensitizing muscle to ordinarily subclinical stress, these pathways may cause severe rhabdomyolysis with even little effort.

According to available clinical data, pine bark extract is safe when taken as prescribed. Both a meta-analysis of 24 randomized controlled trials (RCTs) and a Cochrane systematic review of 27 RCTs found no evidence of severe myotoxicity or rhabdomyolysis, just mild side effects, such as gastrointestinal discomfort [10,11]. Since there have not been any documented cases of pine bark extract-associated rhabdomyolysis as of this writing, this presentation is unique and might have essential pharmacovigilance implications. Reporting such incidents is crucial given the prevalence of supplement usage, even if causality is not established.

Pine bark extract's in vitro effects and reported drug-induced myopathies provide additional support for an association. Certain medications, including statins and antiretrovirals, have been linked to an increased risk of rhabdomyolysis due to mitochondrial dysfunction, disruption of calcium homeostasis, oxidative stress, and apoptosis [12,13,16]. Pine bark extract-treated cells exhibit oxidative stress and metabolic changes, similar to those exhibited by certain medications. Exertion increases oxidative stress and calcium flux, which may interact with supplement-induced risks. Patients who exceed the typical oral dosing of these supplements can present with clinical events and observed effects in their laboratory studies. Patients may not report their supplement usage, and doctors may not inquire regularly, resulting in an underestimation of potential risks.

The widespread use of supplements enhances the case's public health consequences. US surveillance systems indicate that over 23,000 emergency visits are made each year due to supplement-related incidents, and adverse occurrences are underreported [17]. Therefore, clinicians need to inquire about dietary supplements that their patients may ingest to improve their outcomes and prevent the development of adverse conditions. In light of this, even one well-reported instance of extreme toxicity is meaningful.

To our knowledge, there have been no prior case reports of rhabdomyolysis linked to pine bark extract in humans. In the absence of AKI, the patient developed an extreme elevation of CK (>150,000 U/L). The patient was a young, health-conscious individual who sought early evaluation for his symptoms, suggesting that the data were driven by external co-factors rather than solely CK. Given the patient’s findings, a Naranjo score of 3 was calculated, indicating a “possible” adverse drug reaction [18].

Several limitations include the inability to quantify blood levels; validate the precise pine bark extract product, dosage, and purity; and the inability to establish causation. On the other hand, a potential relationship is supported by the time sequence, physiological plausibility, lack of competing hypotheses, and improvement following supplement cancellation.

This novel case provides valuable clinical lessons. Supplements marketed as beneficial may include hidden toxicities, prompting caution while using them. Furthermore, if there is a disproportionate rise in CK after light activity, potential explanations, such as supplements, should be investigated by clinicians. Higher AST and ALT levels in rhabdomyolysis typically reflect CK and may not always imply liver injury. Even when CK levels exceed 100,000 U/L, AKI can be prevented by rapid and aggressive crystalloid hydration [4,5].

## Conclusions

This particular case demonstrates how nutritional supplements, despite their widespread perception as harmless, can produce dose-dependent toxicities that are clinically important. Even after moderate activity, an excessive intake of pine bark extract may reduce the threshold for exertional muscle damage and result in severe rhabdomyolysis. A patient's severe CK elevation (> 150,000 U/L) emphasizes how crucial it is to take supplements into account as possible causes when laboratory abnormalities seem out of proportion to the clinical trigger.

When taking a patient's history, clinicians should specifically ask about supplement use, especially if the patient has high or unexplained CK increases. Early detection, cessation of the suspected substances, and intensive intravenous hydration remain crucial for preventing AKI and improving outcomes. Reporting such incidents is critical for pharmacovigilance and may help direct future research into supplement safety, dose limits, and patient susceptibilities. While causality cannot be established in a single case, the chronological link, biological plausibility, and quick improvement following dechallenge support a possible contribution of supratherapeutic pine bark extract exposure to exertional muscle injury.
