# Correction: Rhabdomyolysis Associated With Excess Pine Bark Extract: A Case Report

**DOI:** 10.7759/cureus.c379

**Published:** 2025-11-18

**Authors:** Arya Kermanshah, Sana Master, Tea Kaceli, Ashmeet Bedi, Garine Kalaydjian

**Affiliations:** 1 Internal Medicine, St. John's Riverside Hospital, Yonkers, USA; 2 Medicine, Lake Erie College of Osteopathic Medicine, Erie, USA

This article has been corrected to remove all references to Pycnogenol as the exact over-the-counter pine bark extract supplement used was unspecified. As a result, all instances of "Pycnogenol" have been replaced with the generic "pine bark extract".

